# Whole-genome assembly of *Babesia ovata* and comparative genomics between closely related pathogens

**DOI:** 10.1186/s12864-017-4230-4

**Published:** 2017-10-27

**Authors:** Junya Yamagishi, Masahito Asada, Hassan Hakimi, Takeshi Q. Tanaka, Chihiro Sugimoto, Shin-ichiro Kawazu

**Affiliations:** 1 Research Center for Zoonosis Control, Hokkaido University, Sapporo, Japan; 2 Global Station for Zoonosis Control, GI-CoRE, Hokkaido University, Sapporo, Hokkaido Japan; 3 Institute of Tropical Medicine, Nagasaki University, Nagasaki, Japan; 4 Faculty of Medicine, Kagawa University, Takamatsu, Japan; 5 National Research Center for Protozoan Diseases, Obihiro University of Agriculture and Veterinary Medicine, Obihiro, Japan

**Keywords:** *B. ovata*, *Babesia*, MinION, Comparative genomics

## Abstract

**Background:**

*Babesia ovata*, belonging to the phylum Apicomplexa, is an infectious parasite of bovids. It is not associated with the manifestation of severe symptoms, in contrast to other types of bovine babesiosis caused by *B. bovis* and *B. bigemina*; however, upon co-infection with *Theileria orientalis*, it occasionally induces exacerbated symptoms. Asymptomatic chronic infection in bovines is usually observed only for *B. ovata*. Comparative genomic analysis could potentially reveal factors involved in these distinguishing characteristics; however, the genomic and molecular basis of these phenotypes remains elusive, especially in *B. ovata*. From a technical perspective, the current development of a very long read sequencer, MinION, will facilitate the obtainment of highly integrated genome sequences. Therefore, we applied next-generation sequencing to acquire a high-quality genome of the parasite, which provides fundamental information for understanding apicomplexans.

**Results:**

The genome was assembled into 14,453,397 bp in size with 5031 protein-coding sequences (91 contigs and N50 = 2,090,503 bp). Gene family analysis revealed that ves1 alpha and beta, which belong to multigene families in *B. bovis*, were absent from *B. ovata*, the same as in *B. bigemina*. Instead, ves1a and ves1b, which were originally specified in *B. bigemina*, were present. The *B. ovata* and *B. bigemina* ves1a configure one cluster together even though they divided into two sub-clusters according to the spp. In contrast, the ves1b cluster was more dispersed and the overlap among *B. ovata* and *B. bigemina* was limited. The observed redundancy and rapid evolution in sequence might reflect the adaptive history of these parasites. Moreover, same candidate genes which potentially involved in the distinct phenotypes were specified by functional analysis. An anamorsin homolog is one of them. The human anamorsin is involved in hematopoiesis and the homolog was present in *B. ovata* but absent in *B. bigemina* which causes severe anemia.

**Conclusions:**

Taking these findings together, the differences demonstrated by comparative genomics potentially explain the evolutionary history of these parasites and the differences in their phenotypes. Besides, the draft genome provides fundamental information for further characterization and understanding of these parasites.

**Electronic supplementary material:**

The online version of this article (10.1186/s12864-017-4230-4) contains supplementary material, which is available to authorized users.

## Background

*Babesia ovata* is one of the bovine *Babesia* species originally isolated in Japan [1]. It is transmitted by the ixodid tick, *Haemaphysalis longicornis*, and is widespread in several East Asian countries [2, 3]. The symptoms that manifest in cattle infected with *B. ovata* are generally mild, including fever and anemia [4]. Adult animals are more susceptible to such parasitic infection than calves, in whom the infection remains subclinical in most cases. In the chronic stage, the infection is subclinical and the parasitic burden of the infected animal is typically submicroscopic, and can only be detected by more sensitive diagnostics such as PCR [4, 5]. However, co-infection with *Theileria orientalis,* which is also transmitted by *H. longicornis,* causes severe anemia and hemoglobinuria [4, 5], which then results in a considerable burden to the livestock industry.

Besides *B. ovata*, several *Babesia* species are known to be infectious to cattle. *B. bigemina* is the most closely related species to *B. ovata* based on phylogenetic and evolutionary analyses using 18S rRNA sequences [6]; however, manifestations are more severe in *B. bigemina* than in *B. ovata* [2, 7]. The *B. bigemina* genome is available in public databases and estimated to be 13.8 Mb in size [8]. *B. bovis* is one of the most extensively investigated *Babesia* species in terms of both biology and bioinformatics. Indeed, its genome was the earliest to be published among the *Babesia* spp. [9] and annotation has been improved with EST and RNAseq data [10, 11]. *B. divergens* genome sequence is also available [8].

The asexual cycle of *Babesia* spp. that replicates in erythrocytes is responsible for pathogenesis and clinical symptoms. Bovine babesiosis caused by *B. bovis* and *B. bigemina* is acute, generally severe, and sometimes life-threatening [7]. Chronic infection has been reported in *B. gibsoni* and *B. divergens* causing canine and bovine babesiosis, respectively [12, 13], and *Theileria* spp. [14, 15]; however, in bovine babesiosis, there is no clear evidence of usual chronic infection, except in cases involving *B. ovata.* Regarding the molecular mechanisms involved in pathogenicity, in cerebral babesiosis caused by *B. bovis* that there is involvement of heterodimeric variant erythrocyte surface antigen (VESA), which is encoded by a multi-gene family, *ves1α* and *ves1β* [16–20]. It has also been proposed that differential expression of the *ves1* gene is involved in the severity of the manifested symptoms [11]. On the other hand, it has been demonstrated in *B. bigemina* that their VESA are coded in *ves1a* and *ves1b,* which are distinct from *ves1α* and *ves1β* [8]. *B. bigemina* also possess the *ves2* gene family, which is distinct from *ves1*, suggesting diversity and evolutionary dynamics of these gene families [8]. Such diversity potentially explains the difference in pathogenicity among these species. Comparative genomic analysis of other *Babesia* spp. should provide an overview of the evolutionary dynamics at the genomic level, including in the *ves* family, which might explain the different characteristics of *Babesia* spp., such as their pathogenicity. Therefore, we aimed to obtain a draft genome of *B. ovata*, with the goal of finding the genomic and molecular bases of its unique phenotypes, namely, chronicity, low pathogenicity, and enhanced pathogenicity upon co-infection.

In terms of methods used for genomic analysis, the development of next-generation sequencing (NGS) offers us a powerful tool. In particular, data produced by Illumina platforms provide us with very high-throughput and high-quality sequence reads. The downside of these sequencing technologies is the short length of their reads. They provide 300-bp paired-end data, at most; this leads to misassembly of repetitive regions and multicopy genes. The latest NGS platforms, such as PacBio or MinION, can overcome such disadvantages by generating long reads based on single-molecule sequencing chemistry, although the sequences of these reads are less accurate than those of the Illumina systems. These less-accurate sequences can be corrected by hybrid assembly, which integrates the two types of NGS, resulting in sets of gene sequences that are of sufficient quality for “omics” data analysis. Since it is known that in silico analysis without experimental data is insufficient to annotate genes and estimate gene models correctly, another hybrid method has been developed to integrate in silico with in vitro*/vivo* data. AUGUSTUS is one such tool that evaluates gene models based on integration of genome and transcriptome data [21].

In this study, we applied the single-molecule next-generation sequencer MinION, together with PacBio RS II expecting high contiguity and accuracy supported by highly reliable Illumina short reads. In brief, 1) reads derived from a MinION and PacBio RS II sequencer were assembled by Canu [22], 2) low-reliability contigs were excluded, and 3) errors were corrected with Pilon using HiSeq reads. In addition, the gene model was estimated based on both information science and an experimental approach supported by RNAseq. Subsequent comparative genomic analyses revealed that the *ves1a* and *ves1b* multigene family, which was originally identified in *B. bigemina*, also exists in *B. ovata*, while the *ves1α* and *ves1β* multigene family originally identified in *B. bovis* was not present. However, *B. bigemina ves1b* did not fully overlap in *B. ovata*, implying the diversified function of *ves1b* in these parasites. In addition, we were able to find *B. ovata*-specific gene families and individual genes, such as extracellular matrix-binding proteins and an anamorsin homolog gene. Their functions remain elusive, but they were identified as potential candidates for having a pivotal function in the pathogenic nature of the parasites.

## Methods

### *B. ovata* Strain and culture

The *B. ovata* Miyake strain was cultured in vitro using purified bovine red blood cells and culture medium M199 supplemented with 40% bovine serum [23].

### Genomic DNA extraction, library construction, and sequencing

Genomic DNA was extracted from *B. ovata*-infected RBCs by the standard phenol-chloroform method [24]. The library for MinION was constructed with a Rapid Sequencing Kit, SQK-RAD003 (Oxford Nanopore Technologies), and then analyzed with two FLO-MIN106 flow cells. Library construction with a TruSeq DNA PCR-Free Sample Prep Kit (Illumina) and 90-bp paired-end sequencing with HiSeq 2000 (Illumina) were performed at BGI JAPAN [25]. Library construction using Lib_Kit (Pacific Biosciences) and sequencing with PacBio RS II using three P6C4 SMART cells (Pacific Biosciences) were performed at Eurofins MWG Operon, Inc. [25].

### RNAseq analysis

*B. ovata* total RNA was extracted from infected RBCs, which were cultured in vitro with TRIzol (Sigma), following the manufacturer’s instructions. Quality and quantity of the purified RNA were validated with Bioanalyzer (Agilent). Library construction for RNAseq was performed as per the instruction manual with TruSeq Stranded mRNA LT Sample Prep Kit (Illumina), and the product was subjected to HiSeq 2500 (Illumina) with the 101-bp paired-end protocol (Illumina).

### De novo genome assembly

Reads obtained from MinION and PacBio RS II were assembled with Canu using genome size = 14 m and default settings for the other parameters [22]. The resulting contigs were examined to subtract possible host contamination and artifacts based on the following criteria. The first criterion was evaluated by relative coding capacity between bovine and *Babesia* parasites (*B. bigemina* and *B. bovis*) evaluated by BLASTX, and coverage less than 10 (mean and standard deviation in the top 10 longest contigs were 29.1 and 4.1, respectively) or if the best hit was against bovine. The second criterion was performed using redundancy among all contigs. The ratio among sequences with similarity to the other contigs evaluated by BLASTN and the whole length in each contig were calculated. The contig with the highest ratio was excluded by iterative analysis until the ratio became less than 90%. The qualified contigs were polished using Illumina reads with Pilon with 26 iterations until a plateau was reached [26].

### Gene model estimation and functional annotation

For gene model estimation, we applied AUGUSTUS version 3.1.0 [21]. To establish trained parameters for AUGUSTUS, webAugustus [27] was utilized, and the required data, the genome sequence (PiroplasmaDB-5.1_BbovisT2Bo_Genome.fasta), and the full-length EST (B.bov.FL-EST.fa) for *B. bovis* were obtained from PiroplasmaDB and DB-AT, respectively [28, 29]. The sequences derived from the RNAseq analysis were mapped onto the assembled draft genome sequence of *B. ovata* with Tophat2. Paired-end reads that failed to be mapped were subtracted by SAMtools. AUGUSTUS in step 1 was performed as follows. An intron hints file with gff format was created by bam2hints script using the Bam file originating from the mapping step. The first AUGUSTUS pass was performed with the trained parameter based on *B. bovis*, the hints gff file, and a parameter file, extrinsic.M.RM.E.W.cfg, which was bundled in the package. The results and hint gff were integrated to obtain an intron dataset and converted to obtain an exon–exon junction database (exex.fa and map.psl) by intron2exex.100.pl script modified from the original intron2exex.pl to accept 100-bp reads. AUGUSTUS analysis in step 2 was performed as follows. The MiSeq RNAseq reads were mapped to the exon–exon junction database with Bowtie2 to obtain spliced reads and their mapped profiles. Unmapped reads were discarded with SAMtools and then the remaining sequences were formatted with SAMmap.pl script with reference to map.psl. The mapped results of the MiSeq reads on *B. ovata* were further filtered to remove N nucleotides with BamTools with operation N filter.txt script in the AUGUSTUS package. The filtered and mapped results were merged with those for spliced reads annotated as above. Another intron file, hints 2, was generated from the merged mapped profile and then a second AUGUSTUS implementation was performed with the trained parameter, hints 2, and extrinsic.M.RM.E.W.cfg.

Functional annotation of *B. ovata* genes, including GO terms, was conducted by Blast2GO [30]. tRNA and rRNA genes in the genome were predicted by tRNAscan [31] and RNAmmer 1.2 web servers [32] with default parameters, respectively.

### Comparative genomics

For the comparative genomic analysis among apicomplexan parasites, we used genome assembly and annotation released in PiroplasmaDB-5.1 for the *B. bovis* T2Bo strain and the *B. microti* RI strain*,* ToxoDB-27 for the *T. gondii* ME49 strain, PlasmoDB-13.0 for the *P. falciparum* 3D7 strain, and GCA_000981445.1 in BioProject PRJEB5046 for the *B. bigemina* BOND strain.

The coding capacities for both tRNA and rRNA in *B. bigemina, B. bovis, B. microti, P. falciparum*, and *T. gondii* were estimated based on the genome sequences by the same method as used in *B. ovata*. Homologs among *B. ovata, B. bigemina, B. bovis, B. microti, P. falciparum*, and *T. gondii* were specified by OMA version 2.1.1 with the default parameters [33]. To examine weak similarity, amino acid sequences coded in *B. ovata* were aligned with those in *B. bigemina*, *B. bovis*, *B. microti*, *P. falciparum*, and *T. gondii* using BLASTP with a threshold of more than 30% identity in more than 30% of the region of the query sequence. To describe the overall relationship among genes in *B. ovata*, *B. bigemina*, and *B. bovis,* an all vs. all BLASTP homology search among them was performed, and gene pairs reciprocally sharing 60% identity over 150 amino acids were selected and then visualized with Gephi (open-source software for exploring and manipulating networks) using a Fruchterman–Reingold layout. Clusters were also specified by Gephi and their representative annotations were referenced from GCA_000981445.1_Bbig001_genomic.gff for *B. bigemina* provided by NCBI and PiroplasmaDB-5.1_BbovisT2Bo.gff for *B. bovis* provided by PiroplasmaDB. The annotation for VESA was acquired from the FTP site provided by the Wellcome Trust Sanger Institute. Reordering of the *B. ovata* assemblies along with *B. bigemina* was performed with Mauve [34]. A dot-plot among the reordered contigs and *B. bigemina* genome was described using YASS [35].

### Expression analysis

Sequence reads from parasite RNA were also examined to obtain expression profiles. These were mapped onto the draft genome sequence along with the predicted gene model with Tophat2 [36]. The information obtained was further mapped to the gene model with HTseq [37] to establish the number of reads covering each gene.

## Results and discussion

### De novo assembly of *B. ovata* genome

A total of 459,000 reads and 389,000 subreads, consisting of 1.6 and 0.9 Gbp, were obtained using MinION FLO-MIN106 and PacBio RS II, respectively (Table 1). In parallel, 12.4 million paired-end reads, consisting of 2.2 Gbp, were obtained using MiSeq.

**Table 1:** Statistics of sequenced reads and contigs

Assembly method	Canu	Canu	Canu	HGAP3
Subtraction of unreliable contigs	Yes	No	No	No
total base pairs	14,453,397	16,393,820	14,312,816	16,481,566
# of contigs	91	228	206	533
N50 contig length	2,090,503	2,093,449	408,732	97,655
N50 contig rank	3	3	11	48
max contig length	3,702,329	3,692,619	1,286,507	529,343
# of MinION raw reads	458,947	458,947	458,947	0
# of MinION total base pairs	1,592,957,533	1,592,957,533	1,592,957,533	0
# of PacBio sub reads	388,599	388,599	0	388,599
# of PacBio total base pairs	954,792,480	954,792,480	0	954,792,480

With Canu assembler, these reads were assembled into 228 contigs comprising approximately 16.2 Mbp. Following subtraction based on redundancy, possible host contamination, and coverage, 91 contigs were qualified (Table 1). The Cane assembler improves the accuracy of the resulting sequences by making consensus in each base; however, it is known that there remain some sequencing errors after this operation. Therefore, we conducted error correction of the HiSeq-derived reads using Pilon. Substitution of 1368 nucleotides, 24,737 insertions and 783 deletions were corrected accordingly, and then the draft genome consisting of 14,453,397 bp was successfully obtained (Table 1). The longest contigs, N_50_, and nN_50_ were 3,702,329 bp, 2,090,503 bp, and the third longest contig, respectively, suggesting this is a draft but nearly complete genome with high contiguity. In this study, we applied MinION sequencer to apicomplexan parasites for the first time. Both MinION and Pac Bio sequencers are known to read out long sequences, which facilitates high-contiguity genome assembly [38, 39]. In particular, it is known that MinION produces ultralong sequences. Indeed, we obtained a 93,434-bp-long read at most in this study and N_50_ was improved from 97,655 by Pac Bio reads only to 2,093,449 by MinION and Pac Bio reads together or 408,732 by MinION only. This suggests the utility of MinION in the assembly of genomes with complicated structures, such as the multiple family genes in apicomplexan parasites.

**Fig. 1:**
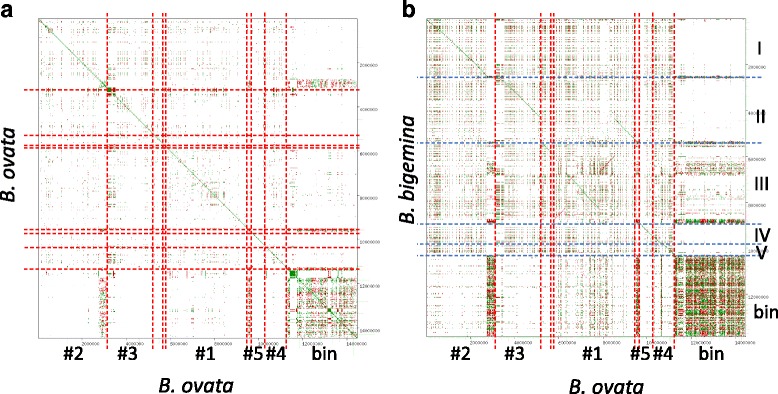
Homology and synteny analysis by dot-plot analysis. Dot-plot comparisons of *B. ovata* against itself (**a**) and *B. ovata* vs. *B. bigemina* (**b**) are presented. The vertical and horizontal dotted lines separate each contig. The Roman numerals on the right side represent the names of contigs in *B. bigemina.* The bin is not a contig but a bundle of short contigs. ID for major contigs in *B. ovata* are also represented. The orders are available in Additional file 4: Table S3

A genome sequence with high contiguity also provides an opportunity for synteny analysis. We aligned the *B. ovata* draft genome sequence on the *B. bigemina* genome and found that structures of chromosomes I, IV, and V in *B. bigemina* were well conserved in *B. ovata.* In contrast, chromosomes II and III were rearranged among them*.* In particular, more rearrangements were observed between chromosome II in *B. bigemina* and the longest contig of *B. ovata* (Fig. 1). This information is useful for future analysis of the evolutionary history among related species.

**Fig. 2:**
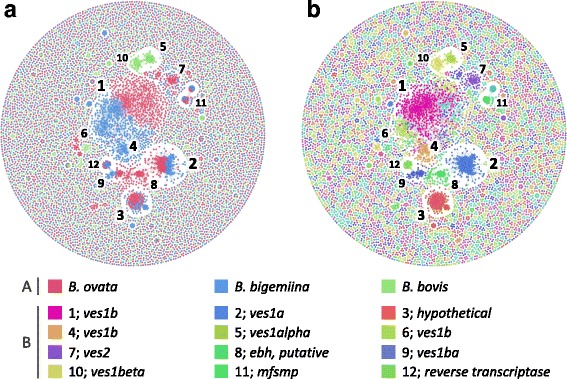
Homolog clustering based on sequence similarity of all genes in *B. ovata*, *B. bigemina*, and *B. bovis*. Each node represents a protein-coding gene in the three parasites. Edges represent similarity between connected nodes. Numbers represent representative cluster ID. **a** The red, green, and blue nodes represent genes in *B. ovata*, *B. bigemina*, and *B. bovis*, respectively. **b** Differences in color correspond to each cluster

### Gene model estimation

For gene prediction, tools based on hidden Markov models such as Glimmer and Genscan are commonly utilized after de novo genome assembly [40, 41]. However, information obtained from such genome sequences is not sufficient to predict authentic gene models and occasionally results in incorrect annotation. Hybrid methods with experimental data are efficacious to avoid such incorrect annotations. In this study, we also applied an additional hybrid strategy, supported by transcriptomics analysis with AUGUSTUS, a tool enabling hybrid annotation with genome and transcriptome data in order to obtain more reliable gene models with functional annotations. The transcriptome of *B. ovata* was obtained from cultured cells by RNAseq using HiSeq 101 paired-end reads. A total of 23.5 Mbp paired-end reads were obtained, which were then mapped on the genome; 60.9% of these were successfully mapped with Tophat2 and the remaining reads were assumed to be host-derived reads or the result of unpaired read mapping. Based on the mapping information, AUGUSTUS predicted 5031 coding regions (CDS), which is almost equal to that for *B. bigemina* (Table 2). In addition, tRNAs and rRNAs were identified with tRNAscan [42] and RNAmmer, respectively, with 64 tRNAs encompassing 20 amino acids, six 5S rRNAs, three 18S rRNAs, and four 28S rRNAs, which is consistent with the most phylogenetically closely related *Babesia* spp., *B. bigemina,* and *B. bovis* (Table 2).

**Table 2:** comparative analysis of genome, gene and homology among representative apicomplexan parasites

	*B. ovata* ^a^	*B. bigemina* ^b^	*B. bovis* ^c^	*B. microti* ^d^	*P. falciparum* ^e^	*T. gondii* ^f^
Genome size (bp)	14,453,397	13,840,936	8,179,706	6,392,438	23,332,831	65,668,596
# of coding genes	5031	5079	3706	3494	5542	8322
# of tRNA	64	46	69	44	76	184
# of 5S rRNA	6	6	9	2	3	47
# of 18S rRNA	3	3	3	2	5	44
# of 28S rRNA	4	3	3	2	6	32
# of B. ovata ortholog (OMA)	N/A	3142	2312	1243	819	693
# of B. ovata homologs (blastp)	N/A	4311	3084	1856	1630	1836

^a^DDBJ accession number BDSA01000001–01000091

^b^GenBank assembly accession number GCA_000981445.1

^c^Registration in PiroplasmaDB reserase 5.1

^d^Registration in PiroplasmaDB reserase 5.1

^e^Registration in PlasmoDB reserase 13.0

^f^Registration in ToxoDB reserase 27

### Functional annotation

Functional identification of the predicted genes was performed with Blast2GO. This provided putative annotation, Gene Ontology (GO), Enzyme Commission (EC) numbers, and Inter Pro Scan results (Additional file 1: Table S1). Based on the analysis, 1371 genes were predicted as hypothetical proteins, whereas the remaining 3660 genes were functionally annotated. In addition, GO and EC identities were assigned to 4305 and 587 genes, respectively. This information was included in a gff file (Additional file 2: Bovata.genome.7.1.gff3).

### Comparative genomics

Next, we performed comparative genomic analysis among typical apicomplexan parasites, *B. bigemina, B. bovis, B. microti, P. falciparum*, and *T. gondii*, based on amino acid similarity using OMA and BLASTP (Table 2). Both sets of genome-wide results suggested that *B. ovata* and *B. bigemina* were the most closely related species, which is consistent with previous studies based on individual genes [2, 43].

Subsequently, we focused on multiplexed gene families among *B. ovata, B. bigemina*, and *B. bovis*, such as the variant erythrocyte surface antigen (VESA). The VESA protein of *B. bovis* is encoded by multiple copy *ves1α*, *ves1β*, and much shorter *ves2* family genes. The heterodimer of VESA 1a and VESA 1b is responsible for antigenic variation of the parasite [19, 44]. Moreover, *B. bovis* VESA is proposed to be involved in cerebral babesiosis via its ability to adhere to host endothelial cells [20]. Comparative analysis among *B. bovis* strains also suggested that VESA is related to the pathogenicity of the parasite [11]. In our study, *B. bovis ves1α* and *ves1β* were also clearly differentiated into clusters #5 and #10, respectively (Fig. 2b, Additional file 3: Table S2). These clusters were *B. bovis*-specific and neither *B. ovata* nor *B. bigemina* genes were included in them, consistent with a previous study of the *B. bigemina* genome [8]. Instead of *ves1α* and *ves1β* forming VESA, it is thought that *B. bigemina* has *ves1a* and *ves1b* repertoires [8]. Regarding *B. bigemina ves1a*, this is featured in a cluster (#2) together with *B. ovata* genes, suggesting that the *B. ovata* genes were *ves1a* (Fig. 2, Additional file 3: Table S2). However, in the detailed analysis, *B. ovata* genes and *B. bigemina* genes were discriminated into two sub-clusters, suggesting that they were not simple ortholog pairs between *B. ovata* and *B. bigemina* (Fig. 2c). This implies that orthologs from the common ancestor multiplied independently in each ancestral parasite lineage over the course of evolutionary history, as suggested previously [8], which might be a process that is still underway. Regarding *ves1b*, there were three clusters containing *B. bigemina ves1b* (#1, #4, and #6). Among them, #4 and #6 were *B. bigemina-*specific (Fig. 2a and b). In contrast, #1 consisted of both *B. ovata* genes and *B. bigemina* genes (Fig. 2a and b). Namely, *B. bigemina* had more diversified *ves1b* genes than *B. ovata*. Besides, cluster #1 was also discriminated into sub-clusters corresponding to *B. ovata* genes and *B. bigemina* genes, the same as cluster #2 for *ves1a* (Fig. 2d). The other VESA-like gene family, *ves2,* is specified in *B. bigemina* [8]*.* This lacks the C-terminal transmembrane motif and GPI anchor signal [8]*.* In *B. bigemina*, 116 genes were assigned as *ves2*, and 18 of these were included in cluster #7 together with 42 *B. ovata* genes (Fig. 2, Additional file 3: Table S2). Other *B. bigemina ves2* formed clusters #24, #31, and #32 and they were almost all *B. bigemina-*specific (Fig. 2, Additional file 3: Table S2). In contrast, *B. ovata* exhibited specific gene clusters, such as #8 involving extracellular matrix-binding protein genes (*ebh*), #13 with extracellular matrix-binding proteins including spectrin repeats, and #15 for an additional *ebh* (Fig. 2, Additional file 3: Table S2). Most of the *ebh* genes were predicted to have transmembrane domains and metal ion-binding proteins (GO: 0046872) (Fig. 2, Additional file 1: Table S1 and Additional file 3: Table S2).

In addition, we observed multiplication of six-cysteine (6-Cys) domain-containing proteins (IPR010884) unique to *B. ovata* and *B. bigemina* and corresponding to cluster #3 (Fig. 2, Additional file 1: Table S1 and Additional file 3: Table S2). Most of them possessed signal peptides, implying that they were secreted proteins. In *B. bovis*, 10 6-Cys domain-containing proteins were identified and predicted to be either secreted or surface membrane-bound and whose expression may be stage-specific [45, 46]; however, cluster #3 and the *B. bovis* 6-Cys genes did not overlap with each other. In *Plasmodium*, sexual stage-specific surface antigen *Pfs48/45* is also a known 6-Cys protein [47]; however, none of the *Babesia* 6-Cys genes showed homology to *Pfs48/45*. As observed here, most of the specified gene families were related to cell-surface expression. It was not surprising to predict that surface proteins are involved in pathogenicity in general. Indeed, it is reported that VESA, PfEMP1, and VSG, as major antigens involved in the immune evasion of *Babesia*, *Plasmodium*, and *Trypanosoma*, respectively, are involved in this process [11, 48, 49]. In this analysis, we observed that *ves1a* and *ves1b* were shared between *B. ovata* and *B. bigemina*, but divided into subclusters. This was more pronounced in the case of *ves1b*. *B. ovata*-specific gene amplification was also observed in *ebh* and others. The acquired diversity potentially explains the differences in phenotype, including in pathogenicity.

In parallel, we also focused on individual unique genes encoded in the *B. ovata* genome; 1788 and 420 genes have no orthologs and showed no similarity to any of the representative apicomplexan parasites, based on OMA- and Blast-based analyses, respectively (Additional file 1: Table S1)*.* For most of these *B. ovata*-specific genes, the function was unclear, except for some such as a GCN5-related N-acetyltransferase (GNAT) family protein (BOVATA_003400) (Additional file 1: Table S1). The GNAT family proteins are known to have histone acetyltransferase (HAT) activity [50]. Those identified in *T. gondii* and *P. falciparum*, TgMYST and PfMYST, have also been demonstrated to exhibit HAT activity and to be involved in gene expression [50, 51]. These genes are functional homologs of BOVATA_003400; however, they did not exhibit significant sequence similarity. Hence, this gene might be associated with critical functions of the parasites via transcriptional regulation or degradation of effector chemicals created by their hosts. tRNA-dihydrouridine synthase (BOVATA_012420) and thioredoxin domain-containing proteins (BOVATA_013620) were identified as other *B. ovata*-specific genes, for which there are functional homologs in other apicomplexan parasites; however, no significant sequence similarity was identified. Therefore, their functional involvement in phenotypes is much more elusive.

The other category of distinct genes in *B. ovata* involves those that are conserved across the apicomplexan species, except in *B. bigemina* (Additional file 1: Table S1). The associated symptoms are among the clear differences between *B. ovata* and *B. bigemina*, and these *B. ovata*-specific genes have the potential to explain such differences. For example, the anamorsin homolog (BOVATA_026810) belongs to this category. Human anamorsin is known to be involved in the suppression of apoptosis in hematopoietic cells [52, 53]. Hence, the homolog in *B. ovata* may also prevent the degradation of hematopoietic cells by the suppression of apoptosis. In contrast, upon *B. bigemina* infection, which lacks such a homolog, severe anemia might result. Mitochondrial import inner membrane translocase subunit (BOVATA_040550) and subtilisin-like protease (BOVATA_022090) also belong to the same category; however, associations among the genes and the differences in phenotype remain elusive.

### Transcriptome of *B. ovata*

Based on RNAseq reads, we obtained transcript frequency data for *B. ovata* (Additional file 1: Table S1). Actin (BOVATA_041600) and ribosomal-related proteins (BOVATA_006170, BOVATA_029000, BOVATA_030990, and BOVATA_020930) were included in a group of highly transcribed genes, as expected. Some highly transcribed *B. ovata-*specific genes were also observed, like hypothetical protein (BOVATA_015540) and methyltetrahydrofolate-homocysteine methyltransferase (BOVATA_026400). These proteins could be potential targets as diagnostic antigens. Besides, highly transcribed putative merozoite surface glycoproteins (BOVATA_028710 and BOVATA_028720) might be potential targets as vaccines. Additionally, BOVATA_021460, which has an ap2 domain most similar to that of *Plasmodium* AP2-G and is highly similar (87% amino acid identity over the entire sequence) to the *B. bigemina* AP2 gene (BBBOND_0104820), was highly transcribed. This implies a potential role of the gene in stage-specific transcription, as reported in other *Plasmodium* parasites [54, 55]. Future comparative transcriptomics in vivo and in vitro*,* with or without *T. orientalis* co-infection, and investigation of intermediate stages, such as acute and chronic infection in ticks, should provide a deeper understanding of babesiosis.

## Conclusions

In this study, we succeeded in obtaining a nearly complete genome, established gene models, and conducted functional annotation by integrating three NGS platforms, MinION, PacBio RS II, and Miseq. We also developed hybrid gene model annotations with genomic and transcriptomic data. By performing comparative genome analysis, we found limited diversity in *ves1b* and *B. ovata*-specific expansion of *ebh* genes, together with a number of *B. ovata*-specific genes such as an anamorsin homolog, which is potentially involved in hematopoiesis in infected hosts. We suggest that the involvement of these genes in the unique phenotypes of *B. ovata* should not be overlooked, even though in the future, these candidates must be examined to verify our hypothesis, taking advantage of the available gene manipulation tools for this parasite [56].

## Additional files

Additional file 1: Table S1. Functional annotation of *B. ovata* genes. Seq_name: Name of amino acid sequence corresponding to annotated genes. RPKM: Normalized mapped RNAseq reads. Columns K to O: Gene ID of orthologs identified with OMA. Columns P to T: Existence of homologs in each species. 1 and 0 represent with and without homologs, respectively. Regarding the other terms in the header, see the Blast2GO publication. (XLSX 1037 kb)

Additional file 2: Annotation with gff format. (GFF3 3895 kb)

Additional file 3: Table S2. Homology clustering. Cluster ID: Cluster ID. Gene: Genes constituting the cluster. Cluster size: Number of genes in the cluster. Annotation: Annotation of the gene. VESA: Appendix annotation for VESA genes. (XLSX 438 kb)

Additional file 4: Table S3. List of contig ID aligned with *B. bigemina* genome. (TXT 1 kb)

## Abbreviations

CA: Celera assembler
CDS: Coding region
*ebh*: Extracellular matrix-binding protein genes
EC: Enzyme Commission number
EST: Expressed sequence tag
GNAT: GCN5-related N-acetyltransferase
GO: Gene Ontology
NGS: Next-generation sequencing
VESA: Variant erythrocyte surface antigen
